# Editorial: Smart Stimuli-responsive Biomaterials for Programmed Drug Delivery

**DOI:** 10.3389/fbioe.2023.1222034

**Published:** 2023-05-22

**Authors:** Seyed Morteza Naghib, Hamid Reza Garshasbi, Ali Maleki, Yasser Zare, Kyong Rhee

**Affiliations:** ^1^ Nanotechnology Department, School of Advanced Technologies, Iran University of Science and Technology, Tehran, Iran; ^2^ Catalysts and Organic Synthesis Research Laboratory, Department of Chemistry, Iran University of Science and Technology, Tehran, Iran; ^3^ Biomaterials and Tissue Engineering Research Group, Department of Interdisciplinary Technologies, Breast Cancer Research Center, Motamed Cancer Institute, ACECR, Tehran, Iran; ^4^ Department of Mechanical Engineering (BK21 Four), College of Engineering, Kyung Hee University, Yongin, Republic of Korea

**Keywords:** smart, stimuli-responsive polymer, biomaterials, drug delivery, programmed release system

The nature of traditional chemotherapy payloads frequently results in substantial adverse effects on healthy tissues since they are not tumor-specific. To change the therapeutic qualities of medications and make them safer and more efficient, several drug delivery systems (DDSs) were created. For maximum effectiveness, high levels of patient compliance, and minimal adverse effects, local medication administration is a crucial strategy that lowers systemic drug exposure.

Smart drug delivery systems (SDDSs) have drawn much interest and opened the door for more successful patient care. The technique that prevents the payloads from being released before they reach the target spot distinguishes SDDSs with stimuli-responsive properties. The precise location of the cargo release is established by the triggered release, which results from changes in the nano/microcarrier chemistry and structure in response to endogenous and/or external stimuli.

The high surface-to-volume ratio, low density, and high hydrophilicity of halloysite nanotubes (HNTs)-polymer composites and the ease with which they may be disseminated in hydrophilic biopolymers have drawn considerable interest. Additionally, it has been proven that they can control the release of medications and carry enough of them. According to research, a gelatin-based scaffold containing a halloysite nanotube (HNT) can serve as a drug carrier. At the same time, zoledronic acid (ZA) sustains release. According to earlier research, administering ZA intravenously has serious drawbacks. However, the goal of the current study is to lessen the negative consequences of local delivery by focusing on the benefits of its osteogenesis. The proliferation of the human adipose stem cells (hASCs) was efficiently increased by the ZA, as demonstrated by the seeding of hASCs on the ready scaffolds. Abdulahy et al. findings suggest that the HNTs-loaded Gelatin scaffold may be able to regulate ZA release and localize its distribution to the defect location while simultaneously enhancing the mechanical and osteogenic potential of gelatin-based scaffolds (Abdulahy et al.).

There are no new, effective treatments for acute lung injury (ALI), which has a significant death rate. The expression of zinc finger E-box binding homeobox 1 and 2 (ZEB1/2), which is positively connected with the development of pulmonary fibrosis, is high in the early stages of ALI. Weng et al. created a nanoscale porphyrin metal-organic (ZPM) framework based on Zr (IV) to deliver tiny interfering ZEB1/2 (siZEB1/2) to treat early lung fibrosis during ALI. Additionally, the ZEB1/2 silencing resulted in higher E-cadherin and lower -SMA levels. An effective non-viral vector technology to distribute siRNAs to treat early lung fibrosis during ALI was the nano-ZPM system (Weng et al.).

An efficient method for targeted cancer therapy with little to no harm is to create nanotechnology-based gene delivery to bladder tumor locations. The c(RGDfK)-MSN NPs, which are mesoporous silica nanoparticles modified with c(RGDfK)-PLGA-PEG, were developed to simultaneously deliver siPD-L1 and miR-34a to bladder cancer cells and tissues. However, in the T24 cells and T24 mouse model, c(RGDfK)-MSN NPs may concurrently downregulate PD-L1 expression, upregulate miR-34a and increase anti-tumor activities *in vivo* and *in vitro*. Shahidi et al. findings provide fresh recommendations for enhancing focused therapy approaches with clear molecular goals for treating bladder cancer (Shahidi et al.).

Increased water solubility and bioavailability of curcumin are produced by its encapsulation in a nanoniosomal delivery system, which also boosts radiosensitivity. With irradiation, the curcumin-containing nanoniosome (Cur-Nio) can increase radiosensitivity. To evaluate cytotoxicity and apoptosis, breast cancer cells were treated with various radiation dosages and different concentrations of free curcumin and Cur-Nio. Additionally, compared to cells treated with pure curcumin, the rate of cytotoxicity and apoptosis in the combination of irradiation and curcumin-containing nanoniosomes was much greater. Afereydoon et al. results suggest that Cur-Nio pre-treatment as a radiosensitizer promotes irradiation-induced breast cancer cell death and is an efficient method to improve the efficacy of breast cancer therapy (Afereydoon et al.).

Using polycaprolactone (PCL) and chitosan (CS), Mosallanezhad et al. created wound dressings. Curcumin (Cur) and zinc oxide nanoparticles (ZnO) were embedded as antibacterial agents in PCL/CS electrospun nanofibers, and various features, including shape, physicomechanical, interaction with water, antibacterial effectiveness, and *in vitro* tests, were examined. The maximum water vapor transformation rate was achieved thanks to ZnO nanoparticles’ facilitation of nanofibers’ contact with water. Effective bacterial growth inhibition was achieved by incorporating CS, ZnO, and Cur together. *In vitro*, tests have revealed that a high Cur concentration reduces cell survival and attachment. The results from the constructed nanofibrous scaffolds showed they have the right qualities to use as a wound bandage (Mosallanezhad et al.).

This study compared the effects of Tween-80, Tween-60, cholesterol, and dioleoyl-3-trimethylammonium propane (DOTAP) on nanoscaled niosomal structures. Compared to free-dispersed Cur, the results showed that Curniosome had better cytotoxic action against cancer cells. Cur-niosome often demonstrates a sizable buildup of excellent anticancer characteristics. Similarly, miR-34a-niosome was more harmful to tumor cells than in its free form. The anticancer effects of gene/drug delivery were studied in the 4T1 xenografted Balb/C mouse tumor model. Considering this, it was determined that encapsulating genes in the nanoniosomal delivery system is a potential approach for curing cancer cells (Abtahi et al.).

Injectable, thermoresponsive hydrogels based on chitosan and silk fibroin have been created using the improved crosslinker system and new gelling agent combinations of glycerophosphate and sodium hydrogen carbonate as a delivery system for hepatocytes in cell therapy. Hydrogel scaffolds with the ideal gelling duration and pH were created by adjusting the polymer-to-gelling agent ratio and using a chemical crosslinker. Chitosan is neutralized by adding sodium hydrogen carbonate while retaining its thermoresponsive properties and having its glycerophosphate content drop from 60% to 30%. Hydrogel’s mechanical qualities are improved by genipin without shortening the gel time. Materials containing genipin have a low swelling ratio, about six, compared to those without genipin, which have a swelling ratio of eight, because of their stable microstructure and reduced amine availability. Silk fibroin works well as a degradation inhibitor in formulations, including silk, since it degrades more slowly than chitosan. The optimized samples all revealed a lack of hemolytic properties. Compared to HepG2 grown alone, urea levels are greater in the encapsulation state. According to all the studies, the optimized system may be a good option for liver regeneration (Gholami et al.).

Given its distinct structural characteristics and promising biocompatibility, the Cu-BTC framework has lately attracted much interest as a potential drug carrier for cancer treatment. However, its inherent incapability for medical imaging may restrict its bioapplications; Using Fe_3_O_4_ nanoparticles as an imaging agent and porous isoreticular MOF [Cu_3_(BTC)^2^ as a drug carrier], a magnetic nano/microscale MOF that addresses this Research Topic has been effectively created by Gharehdaghi et al. With a good pH-responsive drug release, the produced magnetic MOFs have a high loading capacity (40.5%) toward the model anticancer DOX. Clinical applications are anticipated for the synthesized magnetic nano/micro composite. It may also be used as a platform for pH/GSH/photo-responsive nanocarrier and photoactive antibacterial treatment (Gharehdaghi et al.).

